# A Western Veterinary Journal

**Published:** 1883-04

**Authors:** 


A Western Veterinary Journal.—The *United States Veterinary Monthly* is the title of a new veterinary journal recently established in Chicago. There is a dash and *élan* about it which reminds us of the brilliant locomotion of Artemus Ward's kangaroo, "It would please you," said Artemus, "to see the little cuss jump." We congratulate our cotemporary upon its fine appearance. Its editors evidently wish to give it a popular character which will make it attractive to all stock owners. This is legitimate. We only object to its taking the name of a scientific profession, while all the time it aims to be really a non-technical and popular journal. But we wish it success so long as it honestly tries to foster an interest in veterinary science, as it so far seems to be doing.

